# Environmental Information Disclosure, Digital Transformation, and Total Factor Productivity: Evidence from Chinese Heavy Polluting Listed Companies

**DOI:** 10.3390/ijerph19159657

**Published:** 2022-08-05

**Authors:** Hongnan Liu, Weili Liu, Guangchun Chen

**Affiliations:** 1 China Center for Special Economic Zone Research, Shenzhen University, Shenzhen 518060, China; 2 School of Economics and Management, Guangdong Technology College, Zhaoqing 526100, China

**Keywords:** environmental information disclosure, digital transformation, innovation incentive, financing constraints, total factor productivity

## Abstract

Environmental information disclosure, as a new environmental regulatory model, is important for achieving collaborative environmental pollution management and sustainable socioeconomic development. Based on the data of listed firms in China’s A-share heavy pollution industry from 2009 to 2019, this paper empirically tested the impact of environmental information disclosure on the total factor productivity of enterprises and the contribution of digital transformation to this impact. An increase in the level of environmental information disclosure had a significant positive effect on the total factor productivity of enterprises. However, with the increase in digital transformation among enterprises, the effect of environmental information disclosure on total factor productivity improvement is gradually being replaced. The heterogeneity test results showed that the positive effect of environmental information disclosure on total factor productivity changed depending on property rights, firm size, and geographical location. The effect of environmental information disclosure was stronger for non-state firms, large firms, and firms located in the east-central region. Further mechanism tests showed that the effect was induced through innovation incentives and facilitated financing. The above results provide a valuable reference for a comprehensive understanding of the effect of environmental information disclosure on productivity and adjustment by the digital transformation of enterprises.

## 1. Introduction

With increasing international attention on “emission peak” and “carbon neutrality” (“double carbon”), there is an opportunity for environmental information disclosure (EID) and green sustainability development. The new Environmental Protection Law, known as the “toughest ever” in China, was officially implemented in January 2015. For the first time, the law sets clear regulations on the content and methods of EID for heavily polluting enterprises. The “Guidance on Strengthening the Construction of Enterprise Environmental Information System” released in the same year further improved the EID system. However, according to the Evaluation Report on Environmental Responsibility Information Disclosure of Listed Companies in China, almost 74.31% among the total of 4418 listed companies in China had not released their environmental responsibility reports, social responsibility reports, and sustainable development reports in 2020 (data resource: Evaluation Report on Environmental Responsibility Information Disclosure of Listed Companies in China (2020)). As enterprises in polluting industries are the main subjects of EID, external policies on environmental and internal development needs could jointly drive enterprises to actively practice the EID system. For enterprises in heavy pollution industries, EID will have a strong “warning effect”. When facing policy impact, the transformation to a cleaner production mode is the most effective choice for enterprises. EID, as an important means for multiple subjects to collaborate in promoting comprehensive environmental pollution management, and how to effectively evaluate their energy conservation and emission reduction performance, is crucial for China to improve the institutional system of green low-carbon economy. Therefore, encouraging enterprises to participate in EID policy is key to improving total factor productivity (TFP) and core competitiveness. In the “double carbon” background, we discuss two main questions: Does EID enhance or inhibit TFP? What is the mechanism by which EID affects TFP?

In addition, intelligent technology is increasingly used in daily production and operation, and the digital transformation of enterprises has become an important feature of the Industry4.0 era. The improvement of digital transformation has made it possible for the public to have more ways to learn about enterprises, and EID has become less unique. So, will digital transformation disrupt traditional environmental disclosure channels? It is important to clear whether digital transformation plays a supportive or inhibitory role in the impact of EID on TFP. The study of these questions will help investors and corporate managers gain more knowledge about environmental policies and environmental governance behaviors, as well as better digital transformation for green and sustainable development.

## 2. Literature Review

The existing literature on EID focuses on two aspects: the influencing factors of EID and its economic effects. The influencing factors of EID are multidimensional. The earliest research from the perspective of social responsibility can be traced to the social performance theory proposed by Wartick and Cochran (1985) [1]. Companies have a social responsibility to disclose environmental information to the public, and they also benefit from EID. Companies use EID to achieve social responsibility and maintain their advantages [2]. The factors that impact the quality or level of EID include two parts: outside factors, including institutional environment [3], political affiliation [4,5], media attention [6], social reputation [7], and public participation [8]; and internal factors, including internal control [9], corporate governance [10], and executive traits [11,12]. Research from the perspective of economic effects suggests that EID can have an impact on financial performance [13,14], financing costs [15,16], business value [17,18], environmental pollution [19,20], green innovation [21,22], and expanding exports [23,24].

To the best of my knowledge, no literature has directly studied the effect of EID on enterprises’ TFP. The closest study to this research is on the impact of environmental regulation on the total factor of an enterprise. Based on the Porter hypothesis, proper environmental policies and effective implementation are required to effectively promote business innovation. Innovation incentives could optimize the enterprise resource allocation structure and promote productivity growth [25]. Following the “Innovation Offsets” theory, some papers suggest that environmental regulation could stimulate innovation, and thus, promote TFP [26]. In contrast, the “Following Costs” theorists argue that environmental regulation increases the cost burden, and thus, is harmful to the TFP of enterprises [27]. In addition, some papers argue that the relationship between environmental regulations and TFP is non-linear [28]. Furthermore, Zhao and Chen (2022) and Lin (2022) investigated the effect of EID on TFP at the macro level, such as the provincial and municipal level, respectively [29,30]. The above studies delve into the debate between environmental regulation and TFP. As a new ecological governance model, EID provides a new way of thinking for environmental regulation. The research in this paper is a useful addition to the research related to environmental regulation and TFP.

Using the data selected from listed companies in heavy-polluting industries, this paper empirically examines the impact of EID on the TFP of enterprises and explores the mechanism of innovation incentive and financing constraints. We also consider the moderating effect of enterprises’ digital transformation on the impact of EID on TFP. Unlike previous studies, the innovation of this paper is mainly reflected in the following three aspects: (1) In terms of the research perspective, it is the first study to determine the impact of EID on the TFP of enterprises based on the data of listed companies in heavy-polluting industries, which is a useful supplement to the existing studies. At the same time, since heavy-polluting industries are more harmful to the environment and their EID level is higher, it is more relevant to select listed enterprises in heavy-polluting industries as the research sample. (2) In terms of research methods, this paper employs a multidimensional fixed-effects model. We identify the role of EID in effect of TFP, which helps enterprises optimize their environmental management strategies and achieve sustainable development. (3) In terms of research findings, this paper fully considers the digital choice problem in the background of the digital economy. We tested the moderating effect of digital transformation on EID and TFP, which provides a reference for enterprises to better grasp the opportunities of the digital economy.

## 3. Mechanism Analysis

### 3.1. Environmental Information Disclosure and Enterprise Total Factor Productivity

According to social contract theory, the essence of a company is contractual association. Beyond economic benefits, enterprises must also act on social benefits. Enterprises that consider maximizing economic benefits as their development goal must face the trade-off between fulfilling social responsibilities and obtaining economic benefits.

From the perspective of social benefits, EID may improve the exposure of enterprises, thus, promoting the understanding of enterprises by the government, the public, and investors [31]. The positive impression and social trust of enterprises promote long-term development. The quality of an enterprise’s EID will significantly impact its environmental reputation [32]. Good social trust helps enterprises maintain their legal status in the market, enhance their commercial credit financing ability, and improve their market competitiveness [5]. Enterprise administrators can avoid adverse selection problems by communicating internal news to the public through EID. For the government, environmental protection is one of the political goals, and enterprises that generate environmental pollution should also be the target of environmental policy implementation. In addition, enterprises with positive EID attitudes help the government carry out environmental protection work, and in return, they receive environmental compensation and legal market status certifications from the government. For the public and investors, EID can build a good social image and promote investor optimism [33,34]. Therefore, EID reflects the extent to which companies take responsibility for the environment and how well they fulfill their environmental protection systems. A good quality assurance mechanism for EID could achieve low-carbon sustainable development in enterprises [35].

From the perspective of economic efficiency, there is an interaction between EID and business performance. The government strengthens the supervision of enterprises’ fulfillment of environmental protection responsibilities through EID, which could put pressure on enterprises’ management costs and may have a negative impact on their development [27]. EID increases the cost of energy savings, technology expenditures, reporting costs, and additional costs for environmental activities. These expenses increase the financial burden on companies. In addition, enterprises act in advance to obtain better environmental performance based on the expectations of government environmental policies [36]. EID strengthens connections with related stakeholders and reduces the disadvantages of information inequality, thus, effectively reducing investment risks and positively influencing innovation activities [21]. There may be stage differences in the incentive effects of green innovation brought about by EID. Enterprises initially require a large capital investment for green innovation, which is reflected in the cost effect mentioned above. Depending on the effectiveness of green innovation, higher productivity improvement can be achieved through innovation compensation effects [28]. The essence of industrial cleaning is the process of high pollution and high energy consumption industries being replaced by low pollution and low energy consumption industries, and this transformation is obviously conducive to energy efficiency. Enterprises gain goodwill advantages, market legitimacy status, financial access, and technology improvement through EID, which have long-term benefits and contribute to TFP improvement. Therefore, this paper proposes the following hypothesis:

**Hypothesis** **1** **(H1).** *EID is beneficial to green and sustainable enterprise development, leading to an increase in TFP*.

### 3.2. Impact Mechanism of Environmental Information Disclosure on the Total Factor Productivity of Enterprises

#### 3.2.1. Innovative Effects of Environmental Information Disclosure

TFP is influenced by many factors, one of which is innovation. Innovation decisions are risky, but successful innovation has considerable economic value for enterprises. For enterprises, the resource allocation capacity is limited, and taking social responsibility requires resources. Therefore, it is important to clarify the relationship between socially responsible EID behavior, innovation output, and TFP. Research shows that EID could “push” enterprises toward green innovation and cause a synergistic effect to promote TFP [28]. Innovation requires social trust, and a good social reputation allows for good credit financing, which could also be translated into an important motivation for enterprise innovation. The EID system allows for the timely detection of internal environmental governance problems and effectively improves environmental awareness. By regulating this behavior, enterprises can promote the development of green technologies, such as energy savings and emission reductions, and achieve low pollution production and green development [20]. Therefore, EID promotes the optimization of enterprise environmental protection technology and environmental management. It is also encouraged by government environmental subsidies, which strongly encourage innovation and a rising TFP. Therefore, this paper proposes a second hypothesis:

**Hypothesis** **2** **(H2).** *EID promotes TFP through the innovation incentive effect*.

#### 3.2.2. Financing Effects of Environmental Information Disclosure

Based on information the transmission theory, there must be different degrees of information asymmetry between parties. In China, enterprises are mainly financed through debt financing from financial institutions, equity financing, and government financing. To achieve advantage enhancement and gain a market position, enterprises may use EID to gain government support and public attention, resulting in optimistic investment decisions by investors. According to Luo et al. (2019), improving the quality of EID significantly reduces the cost of equity financing. From the perspective of banking institutions and private lending, the quality of EID improves the goodwill of enterprises [16]. Enterprises are thus committed to achieving public acceptance of pollutant reduction through various measures, which leads to increased financing. Investors should also consider the balance between economic benefits and social responsibility. Enterprises that perform EID can be identified as more socially responsible, making it possible for enterprises with higher-quality EID to gain more investment advantages. In terms of government, enterprises that respond positively to environmental policies are more likely to receive government-financed facilities, such as environmental project approval, environmental subsidies, material incentives, special funds, and technology loans. This paper proposes the third hypothesis:

**Hypothesis** **3** **(H3).** *EID increases TFP by alleviating financing constraints*.

### 3.3. Environmental Information Disclosure, Digital Transformation, and Enterprise Total Factor Productivity

Digital transformation has become a key technology for enterprise change, which influences enterprise organization management, resource allocation, and market strategy. Information technology is constantly updated and has become a key factor in upgrading the intelligent operations of enterprises [37]. With the digital platform, enterprises can achieve a high standard of EID, which is also an important way for investors to obtain information. For example, a new product development, the “digital twin” technology, which simulates the parameters of products on a digital platform and can be combined with 3D printing technology, has improved R&D efficiency. In manufacturing, as the digital transformation level improves, artificial intelligence and industrial robots gradually penetrate all aspects of production. The incorporation of digitalization, intelligence, and the product life cycle improves production efficiency [38]. In marketing, digital technology allows the development of customized needs for different users to achieve precision marketing. It reduces resource waste, thus, improving marketing efficiency. In management and decision making based on big data information platforms, information can be dynamically transmitted between business departments. Enterprises can make timely responses to market changes and improve enterprise management and decision-making efficiency.

Digital transformation can, on the one hand, bring new development opportunities for enterprises and promote their productivity improvement. On the other hand, with the continuous development of the digital economy and the increasing level of digital transformation of enterprises, it means that the outside world can have more information channels to understand the whole picture of enterprises [39], which may have substitution and crowding out effects on the productivity effects of EID. In addition, the digital transformation of enterprises is a gradual process, and the blind pursuit of digital transformation may pose hidden risks. Some enterprises wrongly regard the proportion of hardware facilities, such as industrial intelligence, as a measure of digital development and do not recognize the important connection between the digital economy and factors such as technological change and operation mode. Such a misguided transformation may lead to slower technological innovation, a low return on investment, and technology dependence in enterprises. The improvement of enterprise competitiveness should establish an efficient digital transformation and correctly understand its connotation. It should also be used as an important strategy to guide enterprise development and promote sustainable development. Therefore, this paper puts forward the fourth hypothesis:

**Hypothesis** **4** **(H4).** *Digital transformation can have substitution and crowding-out effects on the productivity effects of EID, and has a negative moderating effect on the process of the impact of EID on TFP*.

**Figure 1:**
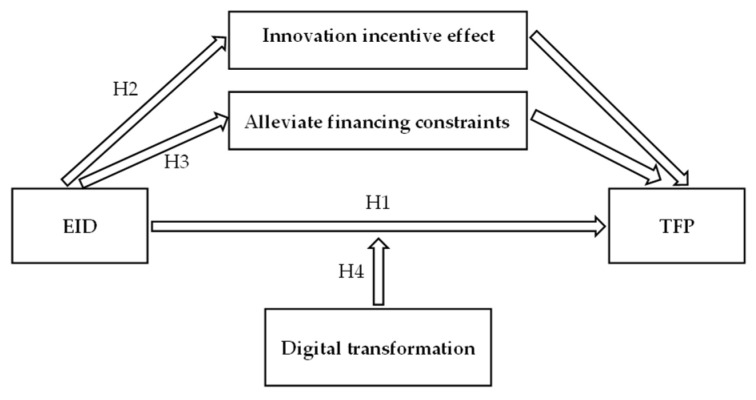
Theoretical framework diagram.

Based on the above theoretical analysis and research hypothesis, we determined the theoretical framework diagram shown in Figure 1.

## 4. Data and Methodology

### 4.1. Data Samples and Sources

Chinese listed companies started to disclose environmental information in 2007; considering the impact of the global financial crisis in 2008 and COVID-19 after 2019, the sample period was selected as 2009–2019. The enterprise data were obtained from the China Stock Market and Accounting Research Database (CSMAR database), and the environmental information disclosure data were obtained from the annual reports of listed companies, the environmental information reports of listed companies, and the social responsibility reports of listed companies.

First, all listed companies that disclosed environmental information during the sample period were collected, and those with incomplete key indicators or seriously missing information, as well as those that were specially treated (marked with ST, *ST, or PT) in that year, were excluded. Then, according to the definition of heavy-polluting industries in the “Guide to Environmental Information Disclosure of Listed Companies” released in 2010 and the “Management List of Environmental Protection Verification Industries of Listed Companies” released in 2008, 16 sub-sectors, including thermal power, iron and steel, coal, chemicals, and building materials, were identified as heavy-polluting industries in this paper. Finally, the sample included 911 listed firms, and 5010 firm-year observations were obtained.

### 4.2. Variable Definition and Data Description

#### 4.2.1. Explained Variable

Most of the current literature uses methods from Olley and Pakes (1996) (OP method) and Levinsohn and Petrin (2003) to measure TFP [40,41]. The OP method uses current investment as a proxy variable for unobservable productivity shocks to overcome the endogeneity problem between productivity and production factors. The LP method is based on the OP method and uses intermediate inputs as a proxy variable for productivity, thus, solving the problem that the OP method cannot be estimated when the amount of investment is zero. In this paper, the TFP measured by the LP method was used as the explanatory variable in the primary regression, while the TFP measured by the OP method was used as a robustness test.

#### 4.2.2. Primary Explanatory Variable

Regarding EID, most current studies use information from annual reports, environmental information reports, and social responsibility reports. Drawing from existing research, the EID level was classified into five primary indicators; namely, environmental management, environmental regulation and certification, environmental information disclosure vehicles, environmental liabilities, environmental performance and governance, and 30 subdivision indicators [42]. Specific descriptions of the metrics of EID are shown in Appendix A Table A1. Finally, we aggregated the scores to determine the level of EID.

According to the Guidelines for Environmental Information Disclosure of Listed Companies, the indicators related to environmental performance, environmental protection expenditure, and the cost of enterprises were defined as hard indicators of EID, denoted as EID_H. These included two first-tier indicators of environmental liabilities and environmental performance and governance and 12 sub-indicators. The indicators related to corporate environmental attitudes, environmental management, and certification, and were defined as soft indicators of EID, denoted as EID_S. These included three first-tier indicators of environmental management, environmental regulation and certification, environmental information disclosure vehicles, and 18 sub-indicators.

#### 4.2.3. Moderator Variables

**Figure A1:**
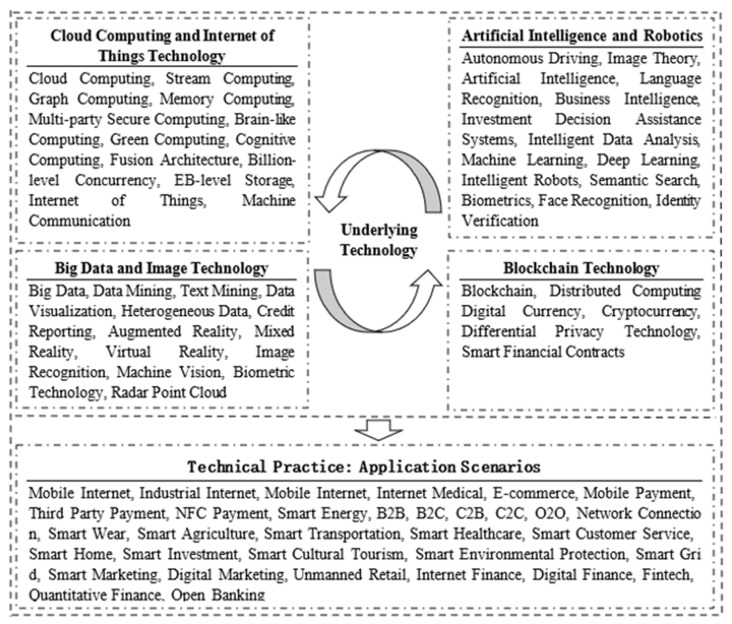
Enterprise digital transformation word frequency diagram.

To calculate the extent of digital transformation, we used Python to extract text from the annual reports of listed companies and counted the frequency of keywords related to digital transformation. Following Wu et al. (2021), we selected two categories of keywords; namely, underlying technology use and technical practice [43]. Underlying technology use mainly consists of four mainstream technology segments, and technical practice focuses on practical application scenarios. The specific word frequencies are shown in Appendix A Figure A1. Finally, based on the feature words in Figure A1, we use Python to search, match, and count the word frequencies of the annual reports of listed enterprises, and then classify the word frequencies of key technology directions and form the final summed word frequencies. The measure of the extent of the digital transformation of enterprises was calculated as ln (total word frequencies +1).

#### 4.2.4. Mediator Variables

The first mediator variable was the innovation incentive level (RD). We followed the approach suggested by Cumming et al. (2016), and used the share of R&D investment in operating revenue [44]. The higher the share, the stronger the innovation incentive.

The second mediator variable was the enterprise financing constraint (WW_index). We adopted the WW_index method to measure the level of corporate financing constraints [45]. The WW_index considers internal financial information in the model while also considering external industry characteristic information, which has broader economic implications. The larger the WW_index, the greater the financing constraints faced by the enterprise.

#### 4.2.5. Control Variables

To avoid the estimation bias caused by omitted variables, the following control variables were added to the model [14]: (1) Business duration (Exist), calculated as ln (current year − year of listing + 1); (2) Investment structure (Inst), calculated as the ratio of the total number of shares held by institutional investors to the total share capital; (3) Enterprise growth (Growth), measured as the growth rate of operating income, calculated as: current year’s operating income/previous year’s operating income − 1; (4) Asset–liability ratio (Lev), calculated as the ratio of total liabilities to total assets; (5) Cashflow ratio (Cashflow), measured by the ratio of net cash flow from operating activities to total assets; (6) Board size (Board), expressed as the logarithm of the number of board members; (7) Industry competitiveness (HHI), as measured by the Herfindahl index, which is the squared sum of sample companies’ market shares in the segmented industry. The descriptive statistics of the main variables are shown in Table 1.

### 4.3. Benchmark Measurement Model

To investigate the impact of environmental information disclosure on enterprise total factor productivity, this paper first constructed the following benchmark model:

(1) $$TFP_{it}=\alpha_{0}+\alpha_{1}EID_{it}+\alpha Control_{it}+\mu_{i}+\omega_{j}+\eta_{t}+\varepsilon_{it}$$

where $TFP_{it}$ represents the total factor productivity of enterprise I in period T; $EID_{it}$ refers to the level of environmental information disclosure of enterprise I in period T; $Control_{it}$ represents a series of control variables; $\mu_{i}$ represents an individual fixed effect; $\omega_{j}$ represents the fixed effect of the industry; $\eta_{t}$ represents time fixed effect; and $\varepsilon_{it}$ is the random disturbance term.

## 5. Empirical Results and Analysis

### 5.1. Benchmark Regression Results

To estimate the effect of EID on TFP, a multidimensional fixed-effect regression was employed in model (1), controlling for enterprise, industry, and year-fixed effects.

As the results are shown in Table 2, with the inclusion of control variables, EID, EID_H, and EID_S coefficients had significantly positive values at the 10, 10, and 5% levels, respectively. This suggests that for enterprises in the heavy-polluting industry, EID significantly improves TFP. An EID is a symbol of an enterprise’s fulfillment of social responsibility, which can have social and economic benefits for enterprises. It contributes to sustainable development and improves TFP growth, which verifies hypothesis 1.

### 5.2. Robustness Test

#### 5.2.1. Replace the Explained Variable

To further verify the impact of environmental information disclosure on corporate TFP, this paper selected all factors calculated by the OP method for robustness testing. From the regression results in columns (1) to (3) of Table 3, when TFP_OP was used as the explained variable, the coefficients of EID and EID_H were both significantly positive at the 5% level. The coefficient of EID_S was positive but not significant. This shows that the improvement effect of environmental information disclosure on the total elements measured by the OP method was mainly reflected in the overall level of environmental information disclosure and the rigid disclosure of environmental information. The test of the substitution variables also demonstrated that environmental information disclosure had a positive impact on corporate TFP.

#### 5.2.2. Add City-Level Control Variables

Since regional characteristic variables may also have an impact on environmental information disclosure and the total factor productivity of firms, to further reduce the bias of omitted variables, this paper further controlled for city characteristic variables [46]. Specifically, the following variables were included: city development level (GDP), measured by city GDP per capita; city size (POP), expressed as the logarithm of total city year-end population; industrial structure (Instr), expressed as the ratio of tertiary industry to secondary industry employees; science and technology investment (Tec), expressed as the ratio of city science and technology expenditures to GDP; education expenditure (Edu), expressed as the ratio of city education expenditure to GDP; and financial development level (Fin), expressed as the ratio of the balance of loans of financial institutions to GDP at the end of the year. The data for city-level control variables were obtained from the China City Statistical Yearbook of previous years.

As shown by the results in columns (4) to (6) of Table 3, the coefficients of EID, EID_H, and EID_S were significantly positive at the 5% level after the inclusion of city-level control variables, and the findings of the study were consistent with the benchmark.

#### 5.2.3. The Method of Instrumental Variables

Companies with higher TFP were more likely to have regulated production processes and stronger environmental management capabilities. They tend to disclose environmental information to increase public trust. An inverse causality between EID and TFP was suspected; thus, a two-stage least-squares method was used. First, following the existing research, the first-lag EID index was selected as the first instrumental variable [47]. Generally, the EID in the previous period had a continuous impact on development in the current period, while the TFP in the current period did not influence EID in the previous period. The endogeneity problem was solved to some extent using the instrumental variable. Moreover, adopting the IV strategy by Faccio et al. (2011), we constructed the mean of other firms’ EID by year and industry as the second instrumental variable [48].

The regression results for the instrumental variables in Table 4 show that the above choices for instrumental variables were valid, as they passed the instrumental variable over-identification test and the weak instrumental variable test. The results from the first-stage estimations in columns (1) and (3) showed that the coefficients were significantly positive at the 1% level, indicating a strong positive correlation between the instrumental and core explanatory variables. Columns (2) and (4) showed that the coefficients of EID were both positive and significant at the 1 and 10% levels, respectively. This indicates that, after controlling for endogeneity, EID still significantly contributed to the improvement of TFP. The results of the above baseline regression were robust, and hypothesis H1 was proven.

### 5.3. Heterogeneity Analysis

#### 5.3.1. Heterogeneity of Enterprise Property Rights

According to the previous theoretical analysis, EID has both economic and social aims. The heterogeneity of property rights has been extensively confirmed in the literature. The quality, motivation, and utility of EID varied among enterprises with different property rights. Tang et al. (2021) showed that EID suppresses the value of state-owned enterprises, while it has a positive effect on the value of non-state-owned enterprises [18]. This paper provides a further analysis of the effect of property rights on the relationship between EID and TFP. Table 5 shows that the coefficients of EID, EID_H, and EID_S were positive but insignificant for the state-owned enterprise sample. EID and EID_S showed a positive effect (at 5% and 1% significance levels) for the non-state-owned enterprise sample, suggesting that EID significantly improves TFP in non-state-owned enterprises, while the effect is limited in state-owned enterprises.

In China’s institutional background, a state-owned enterprise is a political subject directly controlled by the state. It has the advantages of market access, allocation of production factors, access to resources and capital, and higher political legitimacy. Therefore, the main purpose of EID by state-owned enterprises is to respond positively to policy requirements. This reflects their public-interest properties, thereby achieving social impact and political goals. In contrast, non-state-owned enterprises use environmental information disclosure more to achieve business goals. On the one hand, non-state-owned enterprises disclose environmental information to attract the public’s attention and reduce financing costs. On the other hand, they can improve their political legitimacy and market position by conveying to the government that they are actively fulfilling their social responsibilities. Therefore, active EID by non-state-owned enterprises significantly improved TFP, while state-owned enterprises‘ engagement in EID was more of a reflection of the public interest, and the economic effect was not significant, which is consistent with the study by Tang et al. (2021) [18].

#### 5.3.2. Heterogeneity of Enterprise Scale

According to the theory of increasing returns to scale, the larger the size of an enterprise, the more pronounced its economic returns and the stronger its risk tolerance. To explore the influence of enterprise size on the relationship between EID and TFP, the median of total enterprise assets was calculated based on year and industry. Enterprises with total assets greater than the median were defined as “large enterprises”, and those with assets smaller than the median were defined as “small enterprises”. The results are shown in Table 6.

Columns (1) to (3) showed that the regression coefficients of EID, EID_H, and EID_S were significantly positive at the 10% level for large enterprises. In contrast, the coefficients of EID, EID_H, and EID_S were not significant for small enterprises, with a negative value for EID_H, which was as expected. Large enterprises have the advantages of equipment, technology, capital, human resources, and resources, thus, strengthening the TFP improvement effect brought about by EID. Small enterprises are less resilient to risk, and it is difficult to obtain economic benefits from EID. In addition, enterprises are required to make large capital and technological investments to carry out environmental management, which results in environmental policies that may cause unbearable shocks to small enterprises. Therefore, the impact of EID on TFP is insignificant in small enterprises and may even have a negative impact on hard disclosure.

#### 5.3.3. Geographical Heterogeneity of Enterprises

There are economic differences across China, and the differences between regions may have different effects on the relationship between EID and TFP. Based on the geographical location of the enterprises, the samples were divided into eastern, central, and western regions. The results are shown in Table 7.

Columns (1) to (3) showed that the coefficients of EID, EID_H, and EID_S were significantly positive at the 10, 1, and 1% levels, respectively. Columns (4) to (6) showed that, in the central region, EID and EID_H were significantly positive at the 1% level, and EID_S was positive but insignificant. In columns (7) to (9), EID, EID_H, and EID_S were not significant, with a negative value for EID_H. One possible explanation is that the eastern coastal and central regions have higher economic levels and stricter environmental requirements. These companies are more responsive to environmental policies and gain long-term benefits through active environmental disclosure. Enterprises located in the eastern coastal and central region have more capital to make environmental technology improvements and gain the benefits of EID at an earlier stage. In contrast, enterprises in the western region are more backward in economic development and relatively weak in legal regulation. EID is more of a formality and hardly substantially impacts enterprises.

### 5.4. Moderating Effect of the Digital Transformation of Enterprises

To verify the impact of digital transformation on the relationship between EID and TFP, the interaction term between the digital transformation indicator and EID was included in the baseline model (1). The measurement of the digital transformation level was shown in the previous section.

The results are shown in Table 8. Column (1) shows that both EID and Digital were significantly positive at the 5% level, and the interaction term of EID and Digital was significantly negative at the 5% level. Column (2) showed that EID_H and Digital were significantly positive at the 5% level, and the interaction term of EID_H and Digital was significantly negative at the 5% level. Column (3) showed that EID_S and Digital were significantly positive at the 5 and 10% levels, and the interaction term of EID_S and Digital was significantly negative at the 10% level. These results suggest that digital transformation limits the impact of EID on TFP and acts on both hard and soft EID. Hypothesis H4 was validated.

Enterprise digital transformation is an intelligent manufacturing process based on emerging technologies, such as big data, cloud computing, 5G, artificial intelligence, and the Internet of Things. It requires long-term investment and technology accumulation and will not produce immediate benefits. Some enterprises blindly pursue digitalization and intelligence while ignoring the intrinsic connection between the digital economy, technological change, and operation mode, which may adversely affect their business development. In addition, the channels for the public to access environmental information have become more diverse and convenient, which may also have substitution and crowding-out effects on the productivity effects of EID. By taking a scientific approach to digital transformation, we can establish an efficient digital transformation strategy and improve the TFP of enterprises.

## 6. Further Analysis: Mechanism Test

As in the previous theoretical analysis and hypothesis, EID improves TFP through the innovation incentive effect and the improving financing effect. Based on Equation (1), this paper follows Fairchild and MacKinnon (2009) and uses a mediating effect model to test the theoretical mechanism [49]. The model is as follows:

(2) $$Med_{it}=\beta_{0}+\beta_{1}EID_{it}+\beta Control_{it}+\mu_{i}+\omega_{j}+\eta_{t}+\varepsilon_{it}$$

(3) $$TFP_{it}=\delta_{0}+\delta_{1}EID_{it}+\gamma Med_{it}+\delta Control_{it}+\mu_{i}+\omega_{j}+\eta_{t}+\varepsilon_{it}$$

where $Med_{it}$ is the mediating variable, which is the proxy variable for innovation incentives and financing constraints. The measures of indicators were described in the previous section, and the other variables were the same as in the baseline model. Model (2) was the regression of EID on the mediating variables, and model (3) was the regression of EID and mediating variables on TFP.

### 6.1. Innovation Incentive Effect Test

Column (1) of Table 9 shows that the coefficients of EID on RD were significantly positive at the 5% level, indicating that EID can promote enterprise R&D and innovation investment. Column (2) shows that the EID and RD were significant at the 5 and 1% levels, suggesting that EID encourages R&D and innovation expenditure, which in turn improves TFP. Therefore, hypothesis H2 was validated. EID is a manifestation of social responsibility and implies the improvement of a series of environmental protection technologies and management. Although it requires a certain capital investment in the short term, resulting in increased costs, it is beneficial for green and sustainable development in the long run. It enables enterprises’ technological progress and TFP improvement through R&D and innovation expenditures.

### 6.2. Test of Financing Constraints

Column (3) showed that the effect of EID on the WW_index was significantly negative at the 5% level, indicating that EID alleviates financing constraints. In column (4), the coefficient of EID was positive but insignificant, and the coefficient of WW_index was significantly negative at the 1% level, suggesting that EID improves TFP by alleviating financing constraints, thus, validating hypothesis H3. Financing constraints have always been a difficult problem for many enterprises. Daily operations, new product development, production, and sales processes all require a large amount of financial support. EID provides higher exposure, attracts investors’ attention and government support, and largely solves the problem of financing constraints.

## 7. Conclusions

### 7.1. Discussion

Climate change is a global challenge, and countries are actively introducing corresponding policies to address environmental challenges. In 2020, China proposed achieving an “emission peak” by 2030 and “carbon neutrality” by 2060. The requirements for environmental protection have become increasingly stringent, and EID has gradually become a mandatory requirement for companies in heavy-polluting industries. A comprehensive understanding of the social and economic value of EID is important for the sustainable development of enterprises.

In this study, we focus on the impact of EID on the TFP of heavily polluting enterprises based on micro-firm-level data. The results show that EID has a positive impact on the TFP of enterprises. The results of this study support the validity of the “Innovation Offsets” theory [25,26]. In addition, we explored the role of digital transformation in the impact of environmental information disclosure on the total factor productivity of firms by considering the context of China’s developing digital economy. The results show that digital transformation produces crowding-out and substitution effects on EID. As the level of digital transformation of enterprises continues to increase, the contribution of EID to the TFP of enterprises will gradually diminish. Further tests show that the effect is induced through innovation incentives and facilitated financing. Our findings are consistent with the results of Zhao and Chen (2022) and Lin (2022), which are also closer to our research themes [29,30]. Specifically, Zhao and Chen (2022) and Lin (2022) investigate the impact of environmental information disclosure on total factor productivity at the macro level, such as the provincial and municipal levels, respectively. In contrast, we focus our research perspective on the micro-firm level and explore the impact of environmental information disclosure of heavy-polluting firms on their total factor productivity. Moreover, the measurement of EID indicators in our paper is also quite different from their studies.

Above all, this paper is the first to focus on listed companies in the heavy pollution industry to study the impact of environmental information disclosure on the total factor productivity of enterprises, which is a useful supplement to existing studies. Moreover, we also fully explore the digital transformation choices of enterprises in the context of the digital economy and test the moderating effect of digital transformation on environmental information disclosure and total factor productivity, which provides a reference for enterprises to better grasp the pulse of the digital economy.

### 7.2. Main Conclusions

Based on panel data of listed enterprises in the heavy-polluting industry from 2009 to 2019, this paper measured the TFP using LP and OP methods. A multidimensional fixed-effect model was used to examine the effects of EID on TFP and the moderating effect of digital transformation. A series of robustness tests and heterogeneity analyses were conducted to examine the robustness of the findings and the differences in the effects under different conditions. Furthermore, this paper examined the mechanism by which EID affects TFP through a mediating effect model, and the following conclusions were obtained.

(1) EID significantly improves TFP, suggesting that EID should not be considered simply as a cost expenditure but rather as an important way for companies to gain a competitive position and achieve green and sustainable development.
(2) Digital transformation has an inhibitory moderating effect on the impact of EID on TFP. With the development of enterprises’ digital transformation, EID is no longer the only source for the public to understand enterprises’ environmental information. This may cause substitution and crowding-out effects on EID. In addition, some enterprises blindly pursue digitalization and intelligence, while ignoring the intrinsic correlation between the digital economy, technological change, and the operation mode of enterprises. This, in turn, leads to the misplacement of digital resources.
(3) Heterogeneity tests show that the positive effect of EID on TFP varies across property rights, firm size, and geographical location. Specifically, EID has the strongest effect on non-state enterprises, large enterprises, and enterprises located in the east-central region. Therefore, enterprises should fully consider heterogeneous differences and formulate the most suitable strategies when responding to relevant policies.
(4) Further tests on the mechanism suggest that the impacts of EID are transmitted through innovation incentive and financing promotion effects. These results provide useful references for a comprehensive understanding of the productivity effects of EID and the moderating effect of market-oriented transformation, which is conducive to stimulating the vitality of green and sustainable development among enterprises.

### 7.3. Policy Implications

Through previous research, this paper draws on the following policy revelations:

(1) As a reflection of enterprises’ active fulfillment of social responsibility, EID is of great significance to achieve a green economy and sustainable development. The government should improve the EID system and evaluation system to avoid false disclosure and to improve EID quality. A series of incentive policies and public monitoring could help implement environmental policies.
(2) The development of the digital economy has created unprecedented opportunities for enterprises. The rational formulation of a digital transformation strategy, as well as an accurate understanding of the timing of the digital economy, are key to enterprise victory in this new era of technological revolution. Enterprise competitiveness improvement should be based on efficient digital transformation. It is important to correctly understand the connotation of digital transformation and to use it as an important strategy to guide the sustainable development of enterprises.
(3) Enterprises should be fully aware of the heterogeneous characteristics and long-term benefits of EID and should actively fulfill their EID responsibilities. Through the innovative compensation effect, EID covers up-front expenditure. EID improves the financing ability of enterprises and improves TFP. However, the effect of EID on TFP is weak in the western region, where there are small and state-owned enterprises. Therefore, the government should be aware of the regional gap and develop differentiated environmental systems. More support should be given to small enterprises to reduce institutional pressure and financial risks. The effect of EID on TFP was stronger in non-state enterprises, while the effect on state-owned enterprises was not significant. On the one hand, this may be because state-owned enterprises are more conscientious, and the EID incremental effect is not significant. On the other hand, it may be due to the lack of political burden on non-state-owned enterprises, making their strategies more in line with the economic hypothesis of the “rational man”. If we can determine the underlying explanations, we can better achieve a win–win situation.

There are some shortcomings and worthy of in-depth discussion in this study. First, since the companies that make environmental information disclosures represent only a small fraction of the total number of listed companies in China, it was difficult for us to obtain more comprehensive and rich data to conduct the study. With the disclosure of more information and data in the future, this study can be further developed. Second, we did not conduct an in-depth study of why companies are reluctant to disclose environmental information, or what measures companies will take to avoid environmental information disclosure in order to play related games with the government. We believe that as the research continues, these questions will be answered.

## Figures and Tables

**Table 1:** Descriptive statistics of the main variables.

Variables	Observations	Mean	SD	Min	Max
TFP_LP	5010	17.893	1.130	13.381	23.359
EID	5010	10.318	7.830	1.000	37.000
EID_H	5010	5.214	5.331	0.000	24.000
EID_S	5010	5.104	3.060	1.000	15.000
Digital	5010	0.591	0.904	0.000	4.654
Exist	5010	2.135	0.748	0.693	3.332
Inst	5010	0.425	0.245	0.000	1.870
Growth	5010	0.241	1.993	−0.825	84.992
Lev	5010	0.384	0.185	0.011	0.925
Cashflow	5010	0.069	0.066	−0.470	0.600
Board	5010	2.169	0.200	1.099	2.890
HHI	5010	0.071	0.071	0.014	1.000

**Table 2:** Regression benchmark results.

Variables	(1)	(2)	(3)	(4)	(5)	(6)
	TFP_LP	TFP_LP	TFP_LP	TFP_LP	TFP_LP	TFP_LP
EID	0.003 ** (2.071)	0.002 * (1.892)				
EID_H			0.002 * (1.744)	0.002 * (1.675)		
EID_S					0.009 ** (2.565)	0.007 ** (2.179)
Exist		0.175 *** (4.219)		0.175 *** (7.519)		0.175 *** (4.226)
Inst		0.028 (0.658)		0.028 (0.921)		0.028 (0.664)
Growth		0.046 *** (6.993)		0.046 *** (21.609)		0.046 *** (6.974)
Lev		0.598 *** (6.311)		0.597 *** (11.405)		0.596 *** (6.314)
Cashflow		0.654 *** (6.267)		0.652 *** (8.638)		0.656 *** (6.279)
Board		0.272 *** (3.792)		0.271 *** (6.469)		0.274 *** (3.807)
HHI		0.593 ** (2.080)		0.603 *** (3.297)		0.594 ** (2.086)
Constant terms	17.864 *** (1284.571)	16.567 *** (87.093)	17.880 *** (2169.980)	16.579 *** (155.092)	17.846 *** (980.720)	16.550 *** (85.844)
Firm-fixed effects	YES	YES	YES	YES	YES	YES
Industry-fixed effects	YES	YES	YES	YES	YES	YES
Time-fixed effects	YES	YES	YES	YES	YES	YES
Observations	5010	5010	5010	5010	5010	5010
R-squared	0.001	0.193	0.001	0.192	0.003	0.193

Note: * *p* < 0.1, ** *p*< 0.05, *** *p* < 0.01; the value in the brackets is T-value; the regression results are clustered to the enterprise level.

**Table 3:** Robustness test: replacing explained variables and adding city-level control variables.

Variables	(1)	(2)	(3)	(4)	(5)	(6)
	TFP_OP	TFP_OP	TFP_OP	TFP_LP	TFP_LP	TFP_LP
EID	0.001 ** (2.257)			0.003 ** (2.105)		
EID_H		0.001 ** (2.511)			0.003 ** (2.010)	
EID_S			0.001 (0.934)			0.008 ** (2.266)
City control variables	NO	NO	NO	YES	YES	YES
Firm control variables	YES	YES	YES	YES	YES	YES
Constant terms	4.838 *** (94.009)	4.841 *** (95.275)	4.841 *** (92.581)	16.352 *** (27.283)	16.362 *** (37.468)	16.346 *** (27.343)
Firm-fixed effects	YES	YES	YES	YES	YES	YES
Industry-fixed effects	YES	YES	YES	YES	YES	YES
Year-fixed effects	YES	YES	YES	YES	YES	YES
Observations	5010	5010	5010	4868	4868	4868
R-squared	0.092	0.092	0.091	0.196	0.196	0.197

Note: ** *p*< 0.05, *** *p* < 0.01; the value in the brackets is T-value. In order to save space, the specific regression results of the control variables are not reported here, the same as below.

**Table 4:** Robustness test: instrumental variable method.

Variables	(1)	(2)	(3)	(4)
	EID	TFP_LP	EID	TFP_LP
EID		0.038 * (1.882)		0.011 # (1.630)
IV1	0.776 *** (49.168)			
IV2			0.540 *** (34.626)	
Firm control variables	YES	YES	YES	YES
Firm-fixed effects	YES	YES	YES	YES
Industry-fixed effects	YES	YES	YES	YES
Year-fixed effects	YES	YES	YES	YES
Observations	5010	5010	5010	5010
R-squared		0.145		0.178
Kleibergen-Paap rk LM	161.014 ***		58.726 ***	
Cragg-Donald Wald F	2417.509		195.529	

Note: * *p* < 0.1, *** *p* < 0.01; the value in the brackets is T-value; # indicates that it passes the 10.5% significance level; the Cragg-Donald Wald F statistic is used for weak instrumental variable detection, and Stock and Yogo (2005) give the 10% level critical value of 16.38.

**Table 5:** Heterogeneity analysis: differences in the nature of enterprise property rights.

Variables	(1)	(2)	(3)	(4)	(5)	(6)
	SOEs			Non-SOEs		
EID	0.001 (0.426)			0.004 ** (2.475)		
EID_H		0.001 (0.418)			0.004 (1.568)	
EID_S			0.001 (0.289)			0.013 *** (2.994)
Firm control variables	YES	YES	YES	YES	YES	YES
Constant terms	17.477 *** (45.799)	17.481 *** (45.863)	17.475 *** (45.527)	16.564 *** (87.604)	16.588 *** (88.068)	16.535 *** (86.473)
Firm-fixed effects	YES	YES	YES	YES	YES	YES
Industry-fixed effects	YES	YES	YES	YES	YES	YES
Time-fixed effects	YES	YES	YES	YES	YES	YES
Observations	1876	1876	1876	3120	3120	3120
R-squared	0.129	0.129	0.129	0.162	0.160	0.164

Note: ** *p* < 0.05, *** *p* < 0.01; the value in the brackets is T-value.

**Table 6:** Heterogeneity analysis: differences in firm size.

Variables	(1)	(2)	(3)	(4)	(5)	(6)
	Large Scale Enterprise			Small Scale Enterprise		
EID	0.003 * (1.914)			0.000 (0.231)		
EID_H		0.003 * (1.841)			−0.001 (−0.214)	
EID_S			0.008 * (1.800)			0.004 (1.092)
Firm control variables	YES	YES	YES	YES	YES	YES
Constant terms	17.477 *** (0.382)	17.481 *** (0.381)	17.475 *** (0.384)	16.564 *** (0.189)	16.588 *** (0.188)	16.535 *** (0.191)
Firm-fixed effects	YES	YES	YES	YES	YES	YES
Industry-fixed effects	YES	YES	YES	YES	YES	YES
Time-fixed effects	YES	YES	YES	YES	YES	YES
Observations	2388	2388	2388	2476	2476	2476
R-squared	0.133	0.131	0.133	0.182	0.182	0.183

Note: * *p* < 0.1, *** *p* < 0.01; the value in the brackets is T-value.

**Table 7:** Heterogeneity analysis: regional differences.

Variables	(1)	(2)	(3)	(4)	(5)	(6)	(7)	(8)	(9)
	East			Central			West		
EID	0.003 * (1.916)			0.017 *** (2.670)			0.001 (0.151)		
EID_H		0.038 *** (7.580)			0.024 *** (2.627)			−0.001 (−0.310)	
EID_S			0.010 *** (2.689)			0.004 (0.411)			0.008 (0.970)
Control variables	YES	YES	YES	YES	YES	YES	YES	YES	YES
Firm-fixed	YES	YES	YES	YES	YES	YES	YES	YES	YES
Industry-fixed	YES	YES	YES	YES	YES	YES	YES	YES	YES
Time-fixed	YES	YES	YES	YES	YES	YES	YES	YES	YES
Observations	3271	3341	3271	946	946	929	807	807	807
R-squared	0.222	0.467	0.224	0.437	0.438	0.314	0.191	0.191	0.193

Note: * *p* < 0.1, *** *p* < 0.01; the value in the brackets is T-value.

**Table 8:** Moderating effects: the tole of enterprise digital transformation.

Variables	(1)	(2)	(3)
	TFP_LP		
EID	0.003 ** (2.531)		
EID_H		0.004 ** (2.157)	
EID_S			0.009 ** (2.439)
Digital	0.037 ** (2.250)	0.032 ** (2.224)	0.032 * (1.899)
EID × Digital	−0.002 ** (−2.072)		
EID_H × Digital		−0.003 ** (−2.221)	
EID_S × Digital			−0.003 * (−1.794)
Firm control variables	YES	YES	YES
Constant terms	16.560 *** (87.369)	16.573 *** (87.973)	16.541 *** (153.906)
Firm-fixed effects	YES	YES	YES
Industry-fixed effects	YES	YES	YES
Time-fixed effects	YES	YES	YES
Observations	5010	5010	5010
R-squared	0.195	0.194	0.194

Note: * *p* < 0.1, ** *p* < 0.05, *** *p* < 0.01; the value in the brackets is T-value.

**Table 9:** Mechanism analysis: innovation incentives and financing constraints.

Variables	(1)	(2)	(3)	(4)
	RD	TFP_LP	WW_index	TFP_LP
EID	0.015 ** (1.965)	0.003 ** (2.288)	−0.000 ** (−2.083)	0.001 (1.005)
RD		0.025 *** (4.086)		
WW_index				−6.143 *** (−10.138)
Firm control variables	YES	YES	YES	YES
Constant terms	3.394 ***	16.659 ***	−0.979 ***	10.535 ***
	(4.570)	(85.458)	(−63.214)	(16.477)
Firm-fixed effects	YES	YES	YES	YES
Industry-fixed effects	YES	YES	YES	YES
Year-fixed effects	YES	YES	YES	YES
Observations	5010	5010	5010	5010
R-squared	0.013	0.217	0.168	0.383

Note: ** *p* < 0.05, *** *p* < 0.01; the value in the brackets is T-value. In the mediating effect model, all control variables are selected as in the baseline regression, and the results of the control variables are not reported here.

## Data Availability

The data presented in this study are available on request from the corresponding author.

## Appendix A

**Table A1:** Listed companies’ environmental information disclosure indicators.

Disclosure Type	Disclosure Items	Scoring Description
Environmental Management	Environmental Protection Concept	Disclosure: 1 None: 0
	Environmental Goals	
	Environmental Management System	
	Environmental Education and Training	
	Environmental Protection Special Action	
	Environmental Incident Response Mechanism	
	Environmental Honors or Awards	
	The “Three Simultaneous” System	
Environmental Regulation and Certification	Key Pollution Monitoring Units	Disclosure: 1 None: 0
	Pollutant Emission Compliance	
	Sudden Environmental Accidents	
	Environmental Violations	
	Environmental Petition Letter Cases	
	Is Pass ISO14001 Certification	Pass: 1 None: 0
	Is Pass ISO9001 Certification	
Environmental Information Disclosure Carrier	Annual Reports	Disclosure: 1 None: 0
	Corporate Social Responsibility Reports	
	Environmental Report	
Environmental Liabilities	Wastewater Emissions	Quantitative Description: 2 Qualitative Description: 1 None: 0
	COD Emissions	
	SO_2_ Emissions	
	CO_2_ Emissions	
	Soot and Dust Emissions	
	Industrial Solid Waste Emissions	
Environmental Performance and Governance	Waste Gas Emission Reduction and Treatment	Quantitative Description: 2 Qualitative Description: 1 None: 0
	Wastewater Emission Reduction and Treatment	
	Dust and Fume Control	
	Solid Waste Utilization and Disposal	
	Noise, Light Pollution, Radiation and other Governance	
	Cleaner Production Implementation	
